# A Case of Hydralazine-Induced ANCA Vasculitis/Lupus Overlap Syndrome Presenting as Persistent Bicytopenia

**DOI:** 10.1155/crrh/9276592

**Published:** 2025-04-09

**Authors:** Madiha Naqsh Siddiqui, Stephanie Norris

**Affiliations:** ^1^Internal Medicine, University of Michigan, Ann Arbor, Michigan, USA; ^2^Internal Medicine, Trinity Health Ann Arbor Hospital, Ann Arbor, Michigan, USA

## Abstract

**Background:** Hydralazine is a commonly used arteriolar vasodilator that is associated with autoimmune side effects, including drug-induced lupus. A less well-recognized drug-induced vasculitis can be seen, often accompanying drug-induced lupus. This syndrome can cause long-standing vague symptoms, leading to missed diagnoses, and can result in permanent end-organ damage. We describe here such a case of hydralazine-induced vasculitis and lupus overlap syndrome.

**Case Presentation:** An 85-year old male presented with chronic fatigue and weight loss associated with anemia, leukopenia, and acute renal injury in the setting of longstanding hydralazine use. Serologic studies were notable for a positive antinuclear antibody, antihistone antibody, along with anti-myeloperoxidase (MPO) and anti-proteinase 3 (PR3) antibodies. Hydralazine was discontinued, and treatment was initiated with high-dose prednisone. A renal biopsy revealed antineutrophil cytoplasmic antibody (ANCA)-associated focal necrotizing pauci-immune glomerulonephritis. The patient's clinical course was complicated by the development of oral ulcerations and recurrent hydrocele secondary to serositis. Rituximab was then employed without clinical improvement, with eventual progression to end-stage renal disease requiring hemodialysis.

**Conclusions:** This case report helps highlight the vague symptoms that can be associated with hydralazine-induced vasculitis/lupus overlap syndrome. This case will increase clinician awareness for early recognition of such a syndrome, prompting early diagnosis, preventing end-organ damage, reducing hospitalizations and improving quality of life.

## 1. Background

Hydralazine is a commonly used selective arteriolar vasodilator, prescribed often to treat hypertension or heart failure for afterload reduction. Introduced to routine clinical practice in 1951 [1], this medication gained interest given its quick onset of action, lack of major drug-drug interactions, and easy to titrate dosing. Although up to 10% of patients can experience hydralazine-induced lupus (HIL) [2] and lupus-like syndromes, these adverse effects are not routinely considered when prescribing hydralazine. The following case highlights the importance of early recognition of such side effects, as complications such as bicytopenia and end-stage renal disease (ESRD) can lead to significant reduction in patients' quality of life as well as frequent readmissions.

## 2. Objectives

• To recognize the adverse effects of hydralazine, specifically hydralazine-induced vasculitis and lupus
• To outline the natural history and outcomes of hydralazine-induced vasculitis and illustrate patient presentation with emphasis on complications such as bicytopenia and ESRD
• To identify lab abnormalities associated with hydralazine-induced vasculitis and lupus overlap syndrome

## 3. Case Report

An 85-year-old Caucasian male presented with complaints of progressive fatigue, cold intolerance, and loss of appetite leading to a weight loss of approximately 30 pounds over the course of a year. His past medical history was notable for atrial fibrillation, heart failure with preserved ejection fraction, and chronic kidney disease Stage 4. The patient had been taking hydralazine 100 mg three times a day for the past 20 years. He had undergone extensive workup for the above symptoms at an outside hospital; at that time, he was found to be severely anemic and leukopenic, with a hemoglobin level of 6.1 g/dL (reference range: 13.0–18.0 g/dL) and white blood cell count of 1.2 K/μL (reference range: 3.7–11.0), respectively. A peripheral blood smear had shown normocytic anemia along with neutropenia, and iron studies were consistent with iron deficiency anemia. He underwent transfusion of two units of packed red blood cells (RBCs) and intravenous (IV) iron sucrose, with improvement. He was then discharged with a plan for outpatient hematology evaluation.

Upon outpatient follow-up, results of his complete blood count again revealed bicytopenia, with a hemoglobin level of 6.5 g/dL. He underwent a bone marrow biopsy that ruled out leukemia, lymphoma, myelodysplasia, or plasma cell neoplasm, demonstrating a persistent normocytic anemia. Complement testing revealed low C3 and normal C4. In addition, he was noted to have progressively worsening renal function with a creatinine of 2.65 mg/dL (reference range: 0.60–1.30). These results prompted his primary care physician to obtain autoimmune studies. The patient was instructed to discontinue hydralazine. Results of serological testing were as follows (Table 1):

He was then brought to the hospital by his family due to persistent fatigue and for further rheumatologic workup. Physical examination did not reveal livedoid discoloration, periungual erythema, or any other rash. No synovitis was noted in the proximal or distal interphalangeal, metacarpophalangeal, or radiocarpal joints.

Laboratory testing revealed the patient to be in acute renal failure, with 3+ hematuria; he was also found to have a strongly positive antihistone antibody. This raised concern for concomitant hydralazine-induced vasculitis (in addition to HIL). The patient was initiated on high-dose prednisone, 40 mg daily. ANCA testing and testing for inflammatory markers were repeated, with results as follows (Table 2).

**Figure 1:**
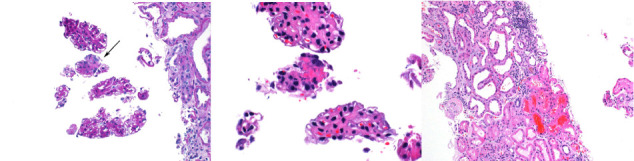
Glomerulus with fibrinoid necrosis [1, 2]. Acute tubular injury with red blood cell casts [3].

The patient reported improvement in his fatigue and weakness on Day 4 of treatment with 40 mg of prednisone; however, renal function continued to worsen with increasing creatinine values. A renal biopsy was obtained, showing focal necrotizing glomerulonephritis, pauci-immune type (Figure 1) consistent with ANCA-associated glomerulonephritis. The patient was then initiated on induction therapy with rituximab in attempts to mitigate further organ damage; this was initiated at 1500 mg IV with plan for a repeat dose in 15 days' time. His clinical course was complicated by the development of multiple small ulcerations on the hard palate, tongue, upper pharynx, and epiglottis, visualized on laryngoscopy. He also developed recurrent hydroceles, believed to be secondary to serositis. However, the patient's renal function stabilized at a creatinine value of 3.7 mg/dL. He was discharged after a 1-month hospital stay, on prednisone 40 mg/d with plan to taper slowly (decreasing dose by 10 mg every 2 weeks). He received his second dose of rituximab outpatient.

The patient was readmitted in 1 weeks' time due to lower gastrointestinal (GI) bleeding while on prednisone. At the time of admission, he was on prednisone 30 mg daily. Nephrology was consulted during this hospitalization due to worsening renal function (noted to have a rising creatinine up to 4.29 mg/dL) and advised increasing the dose to 40 mg/day, with a slower taper on discharge. The patient's creatinine subsequently returned to 3.7 mg/dL, and he was discharged, with a plan to taper prednisone dose by 5 mg every month. He was readmitted again in 1 week's time with shortness of breath and hypoxia. He was noted again to have worsening renal function, now with a creatinine of 6.17 mg/dL. Nephrology advised hemodialysis initiation, with significant improvement in symptoms by the time of discharge (5 days). The patient remained dialysis-dependent secondary to ESRD. After subsequent hospitalizations, the patient and his family ultimately decided to transition the patient to hospice care.

## 4. Discussion and Conclusions

Hydralazine can induce autoimmunity via various mechanisms, including depletion of neutrophilic histone H3K27me3 at PR-3 and MPO, resulting in demethylation leading to gene activation. Additional proposed mechanisms include inhibition of DNA methyltransferase, and reversal of PR3 and MPO gene-slicing [3], leading to expression of concealed antigens. This subsequently activates B- and T-cells, leading to the formation of ANCA autoantibodies [4].

Hydralazine is strongly associated with the causation of drug-induced lupus, carrying a risk of > 5% [5] with a probability of up to 8%–13% [6]. Of those who develop HIL, renal involvement is rare, seen in < 5% of patients [7]. However, hydralazine-induced ANCA vasculitis (HIAV) frequently involves the kidneys, and clinical suspicion for ANCA vasculitis rather than lupus should be maintained when renal failure is the primary manifestation.

A dose-dependent incidence has been reported, with the lowest causative dose being 100 mg once daily [8]. Multiorgan involvement is seen, including necrotizing mucocutaneous lesions [9], polyarthralgias, serositis, diffuse alveolar hemorrhage [10], renal dysfunction with glomerulonephritis, weight loss, and anemia [11]. Laboratory findings and serologies common in HIL and HIAV include C3 hypocomplementemia, ANA, and antihistone antibody [12]. Positive pANCA/anti-MPO is seen more often as compared to cANCA/anti-PR3, with some cases being dual positive. Additionally, antiphospholipid antibodies including anticardiolipin [13] can also be seen; these along with dual ANCA positivity was observed in the case illustrated above. As noted in a study by Santoriello et al. [14], duration of treatment and mean daily dose of hydralazine were at least 1 year and 250 mg/day, respectively. Our patient's case followed this trajectory, with duration of treatment with hydralazine being 20 years and a dose of 300 mg/day.

Management includes cessation of hydralazine. Treatment can be classified into two arms, including induction and maintenance therapy. Induction therapy includes steroids, rituximab, cyclophosphamide, mycophenolate mofetil, and/or plasmapheresis. Maintenance therapy tends to include azathioprine and mycophenolate mofetil. Unfortunately, renal outcomes remain poor, with most patients progressing to ESRD [14] including our patient. High-remission rates are observed regardless of choice of therapy [15].

The clinical index of suspicion for adverse effects associated with hydralazine use should be high in patients on long-term hydralazine therapy. Side effects such as HIAV or HIV can often present with subtle symptoms and vague laboratory findings that can easily be overlooked. As highlighted in the case report above, both HIAV and HIV are associated with a high morbidity and mortality rate despite drug cessation and appropriate treatment. Thus, high-risk patients should be identified, and hydralazine should be substituted with alternative antihypertensive medications.

## Figures and Tables

**Table 1:** Autoimmune serologies and complement levels.

Test	Result
Antinuclear antibody (ANA)	> 1:1280 IU/mL Homogenous and speckled. Reference range: negative, < 1:80 IU/mL

Antineutrophil cytoplasmic antibody (ANCA)	< 1:20 AI Reference range: < 1:20 AI

Anti–double-stranded deoxyribonucleic acid (anti-dsDNA)	Negative, 1 IU/mL Reference range: negative, < 5 IU/mL.

Anti-Smith antibody (anti-Sm)	Negative Reference range: negative

Anti–Sjögren's syndrome–related antigen A (anti-SSA)	Negative Reference range: negative

Anti-Sjögren's syndrome type B (anti-SSB)	Negative Reference range: negative

Anticardiolipin antibody and immunoglobulin M (IgM)	Low-medium positive, 33 MPL U/mL Reference range: Negative: IgM < 12.5 MPL U/mL Indeterminate: IgM = 12.5–20 MPL U/mL Positive: IgM > 20 MPL U/mL

Anticardiolipin antibody and immunoglobulin A (IgA)	Negative, < 9.5 APL U/mL Reference range: Negative: < 20 APL U/mL Positive: > 20 APL U/mL

Anti-cardiolipin antibody and immunoglobulin G (IgG)	Negative, 13.4 GPL U/mL Reference range: Negative: IgG < 15 GPL U/mL Indeterminate: IgG = 15–20 GPL U/mL Positive: IgG > 20 GPL U/mL

Cold agglutinin titer	Negative, < 1:4 Reference range: titer less than 1:40

C3	59 mg/dL Reference range: 75–175 mg/dL

C4	19 mg/dL Reference range: 10–40 mg/dL

**Table 2:** Inflammatory markers, autoimmune serologies, and ANCA levels.

Test	Result
Erythrocyte sedimentation rate (ESR)	43 mm/hr Reference range: Male: 0–10 mm/hr Female: 0–20 mm/hr

C-reactive protein (CRP)	8.9 mg/dL Reference range: 0.0–4.9 mg/L

Antihistone antibody	10.1 units Reference range: Negative: < 1.0 units Weak positive: 1.0–1.5 units Moderate positive: 1.6–2.5 units Strong positive: > 2.5 units

C-ANCA/anti-PR-3	32 AU/mL Reference range: < 1:20

P-ANCA/anti-MPO	118 AU/mL Reference range: < 1:20

## Data Availability

This case report does not include generated data or data from publicly available data sets.
